# Solid Concentration Measurement in Horizontal Gas–Solid Flows: An Adaptive Model Matching Strategy Using Array Capacitive Sensor

**DOI:** 10.3390/mi17070866

**Published:** 2026-07-21

**Authors:** Zengyan Zhu, Dayang Wang, Yan Li

**Affiliations:** 1 School of Internet of Things Engineering, Wuxi University, Wuxi 214105, China; 860546@cwxu.edu.cn; 2 College of Information Science and Engineering, Northeastern University, Shenyang 110819, China; 3 School of Cybersecurity, Wuxi University, Wuxi 214105, China; 860547@cwxu.edu.cn

**Keywords:** gas-solid two-phase flow, array capacitive sensor, horizontal pipeline, BP-adaboost, K-means

## Abstract

The accurate measurement of solid concentration in horizontal gas–solid flows is very important to guarantee production efficiency and process control. However, gravity causes uneven particle distribution, which creates a strong nonlinear relationship between sensor signals and solid concentration. When particle distribution changes, a single linear measurement model cannot provide enough detection accuracy. This paper proposes an adaptive model matching strategy for solid concentration measurement in horizontal gas–solid flows by using array capacitive sensor. The array capacitive sensor works with two excitation modes. The concave-ring excitation mode collects particle distribution information, and the multi-electrode excitation mode obtains solid concentration. These two types of signals build a dynamic matching relationship between particle distribution features and measurement models. In detail, signals from concave-ring excitation are input into the BP-Adaboost algorithm to classify particle distribution states. Multiple linear measurement models between multi-electrode signals and solid concentration are built through K-means clustering. After recognizing the particle distribution type, the Euclidean distance is used to automatically select the corresponding measurement model. A 3D simulation model combining gas–solid two-phase flow and electrostatic field is set up to test the feasibility of the proposed measurement method. Laboratory experiments are also conducted to prove its reliability. The test results show that the method can adapt well to various particle distribution states. Within the solid concentration range of 0.34–14.08%, the average relative measurement error is 4.41%. This method effectively improves the measurement accuracy of solid concentration in horizontal gas–solid two-phase flow.

## 1. Introduction

Gas–solid two-phase flow systems are prevalent in nature and industry. A typical industrial application is pneumatic conveying, which transports powders and granular materials. The reliable, online, and non-intrusive measurement of flow parameters in gas–solid two-phase flow systems is essential for ensuring efficient energy utilization and industrial safety [1,2,3]. Various methods and techniques have been studied and developed for measuring solid concentration and velocity.

Existing gas–solid flows measurement technologies can be generally split into intrusive and non-intrusive categories. Non-intrusive detection avoids disturbing the original flow field and thus has broader application prospects for long-term industrial process monitoring. Typical non-intrusive solutions include acoustic, radiation, optical and capacitive techniques. By contrast, electrostatic probes are intrusive measurement tools that insert electrodes into pipelines and disturb natural particle flow. All the above approaches have been widely adopted for solid concentration and velocity measurement, yet each has distinct inherent advantages and disadvantages determined by their physical principles. For instance, the acoustic method is unsuitable for harsh industrial environments, while the radiation method suffers from problems including radiation leakage risks, complex structures, and high sensor costs. Among these techniques, concentration measurement based on capacitive sensors has attracted considerable attention owing to its merits of simple, reliable structure, low cost, fast response, and wide applicability in industrial scenarios.

In industrial applications, gas–solid flows exhibit significant variability across horizontal, inclined, and vertical pipeline configurations. Among these, particles in horizontal pipelines exhibit more complex flow behaviors due to the perpendicularity between the flow direction and gravitational force. Xiao et al. [4] observed stratified flow as the dominant regime during dense-phase conveying of fine pulverized coal in horizontal pipelines. At the gas-solid interface, coal particles exhibited wave-like axial motion with irregular radial movement, characterized by high turbulence intensity and rapid transience. Through time-sequenced electrical capacitance tomography (ECT) image stacking, Cong et al. [5] visualized slug flow, fluidized flow, slurry flow, and stratified flow in horizontal pneumatic systems. They emphasized that dense-phase coal conveying typically features multiple coexisting flow regimes, which are often dominated by one or two primary regimes rather than a single deterministic regime. As a soft-field detection technique, capacitive sensors’ sensing field varies with medium distribution. When such sensors are externally installed on a circular pipeline, their sensitive field exhibits significant non-uniformity. Thus, capacitance-based two-phase flow measurement is highly dependent on the particle distribution, and the complex gas–solid flows inside horizontal pipelines poses a significant challenge to measurement accuracy.

To address this challenge, researchers have optimized the configuration of capacitive sensors to enhance the uniformity of sensitivity distribution [6,7,8,9,10]. Zhai et al. [6] investigated the effects of geometric structures on sensitivity profiles, demonstrating that the dual-helical electrode configuration can effectively reduce the impact of flow regimes on measurement signals. Li et al. [10] further refined this approach through calibrated instrumentation, designing dual-helical capacitive sensors with optimized geometries that significantly improve the homogeneity of the sensitivity field. Despite better overall uniformity, the dual-helical sensor still faces limitations in achieving highly uniform sensitivity distribution. The sensitivity is higher in the edge regions of the measurement field than in the central region. Another approach involves establishing a relationship between measurement signals and solid concentration based on the identified flow regimes to achieve accurate solid concentration measurement [11,12]. Zhang et al. [11] introduced an integrated instrumentation system for measuring volumetric concentration in biomass/coal/air three-phase flow within pneumatic conveying pipelines. During measurement, flow regimes such as rope flow and stratified flow were first identified through the Hilbert marginal spectrum of electrostatic sensor signals. A dual-regression analysis method was then employed to calculate biomass concentration and pulverized coal concentration. Wang et al. [12] combined electrostatic and capacitive sensors to measure phase concentration in pulverized coal/biomass/air three-phase flow using data fusion. During detection, Mel-frequency cepstral coefficient features were extracted from electrostatic signals to train a continuous Gaussian mixture hidden Markov model, which identified flow regimes including rope flow, dispersed flow, and stratified flow. On this basis, a support vector machine was introduced to establish a concentration information fusion model under the identified flow regimes.

The above studies indicate that the identification of gas–solid flow regimes via various sensing technologies has been extensively investigated and applied. However, the above research mainly focuses on the macroscopic definition and identification for typical flow regimes, such as slug flow, dune flow, laminar flow, and suspended flow. In a gas–solid flow process, various typical flow states transform rapidly and coexist with transitional flow states. For example, slug flow is regarded as a single macroscopic flow regime, but its particle distribution exhibits prominent spatial differences. The particles in the front and rear regions of slugs do not fully occupy the pipeline cross-section, and continuous particle-pouring and pick-up exchange occurs between these dilute transitional regions and dense slug bodies. In such scenarios, the distinct dynamic particle distribution features within the same macroscopic flow regime couple with the non-uniform sensitivity distribution of the measurement domain, resulting in a nonlinear relationship between the sensor output and the actual solid concentration, which affects the solid concentration measurement accuracy.

To address the challenges posed by complex and variable flow regimes to solid concentration detection in horizontal pipelines, this paper proposes an adaptive model matching strategy for solid concentration measurement in horizontal gas-solid flows by using array capacitive sensor. Relying on a single array capacitive sensor, it uses the concave-ring excitation mode to obtain particle distribution information and the multi-electrode excitation mode to measure particle concentration, establishing a dynamic matching mechanism between distribution characteristics and concentration measurement models.

In the measurement process, the concave-ring excitation signal is first subjected to feature extraction, and its spatial and temporal features are used to construct feature vectors, which are then input into the BP-Adaboost algorithm for particle distribution classification. After identifying the distribution type, the corresponding linear measurement model is adaptively matched by calculating the Euclidean distance between the parameter of signal vertical stratification ratio and each cluster center, predetermined using K-means clustering during calibration. Through the synergy of the two algorithms, the nonlinear mapping between sensor signals and concentration is decomposed into multiple linear relationships, effectively improving the measurement accuracy of particle concentration. This study integrates mature technologies with a customized dual-excitation array capacitive sensor tailored for horizontal gas–solid two-phase flow, further constructing a dedicated adaptive model matching framework to eliminate the nonlinear solid concentration measurement errors caused by complex and changeable flow regimes in horizontal pipelines.

To verify its effectiveness, a 3D numerical simulation model coupling gas–solid two-phase flow and an electrostatic field is established, with laboratory physical experiments validating the model’s reliability. By designing different flow conditions, different flow processes are simulated for verification. The experimental results show that the proposed measurement method can stably adapt to different particle distributions and improve the accuracy of particle concentration measurement in horizontal gas–solid two-phase flow. In this work, the developed dual-excitation modes and adaptive model matching framework can dynamically apply corresponding measurement models according to the detected flow states, which reduces concentration measurement errors in horizontal gas–solid flows. Furthermore, the proposed sensing scheme employs a compact electrode design and centers on microscale electrostatic interactions between electrodes and discrete particles. The resulting miniaturized non-intrusive measurement platform delivers a low-cost, practical detection solution for industrial gas–solid two-phase flow monitoring.

## 2. Hardware Design of Array Capacitive Measurement System

### 2.1. Concave-Ring and Multi-Electrode Excitation Modes for Array Capacitive Sensor

**Figure 1:**
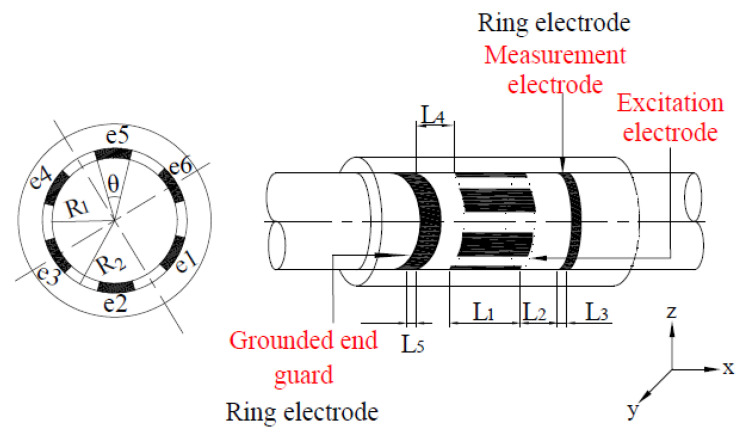
The array capacitive sensor composed of six concave electrodes and two ring electrodes, where *e*1–*e*6 denote the first to sixth concave electrodes, respectively. Other symbols: the length of concave electrode *L*_1_, the length of ring measurement electrode *L*_3_, the distance between ring measurement electrode and concave electrode *L*_2_, and the wrap angle of concave electrode θ, the length of guard electrode *L*_5_, the distance between guard electrode and concave electrode *L*_4_, the inner radius of pipe *R*_1_, the outer radius of pipe *R*_2_.

**Figure 2:**
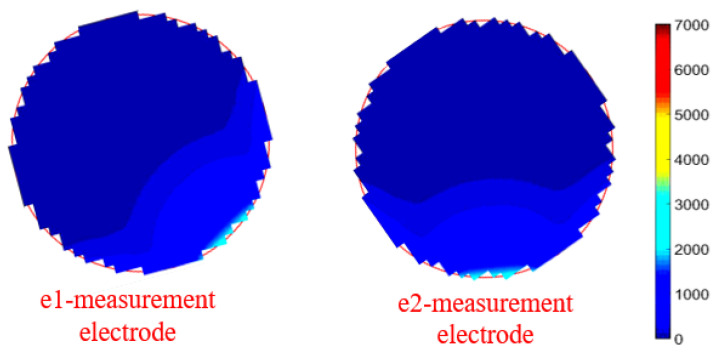
Typical radial sensitivity distribution under the concave-ring excitation mode when the measurement field is filled with air.

In the horizontal gas–solid flows, particles tend to concentrate at the pipeline bottom under gravitational influence, forming a variety of distinct distributions. To effectively detect particle distribution features in horizontal pipelines, this study employs the concave-ring excitation mode based on an array capacitive sensor. The structure of the array capacitive sensor, which comprises six concave electrodes and two ring electrodes, is illustrated in Figure 1. Under the concave-ring excitation mode, the concave electrodes sequentially act as excitation electrodes, while one ring electrode serves as the measurement electrode. The ring electrode at the opposite end of the sensor is grounded and acts as a guard electrode to mitigate fringe effects and noise interference. The sensitivity field distribution within the measurement region under the concave-ring excitation mode is shown in Figure 2. As indicated in Figure 2, the distribution characteristics of the sensitive fields corresponding to all measurement signals in the concave-ring excitation mode are similar, and their high-sensitivity areas are significantly localized.

In horizontal pipelines, particle flows typically accumulate at the pipeline bottom, and their distribution exhibits a certain degree of symmetry in the radial cross-section. By combining this symmetry with the sensitivity distribution in the measurement region, the measurement signal under the concave-ring excitation mode can effectively reflect the local distribution of particle flow. Furthermore, compared to the excitation mode used in traditional ECT measurement systems, the concave-ring excitation mode ensures real-time detection capability for localized information. Consequently, the concave-ring excitation scheme of the array capacitive sensor is selected to detect the localized distribution features of particles in horizontal pipelines.

**Figure 3:**
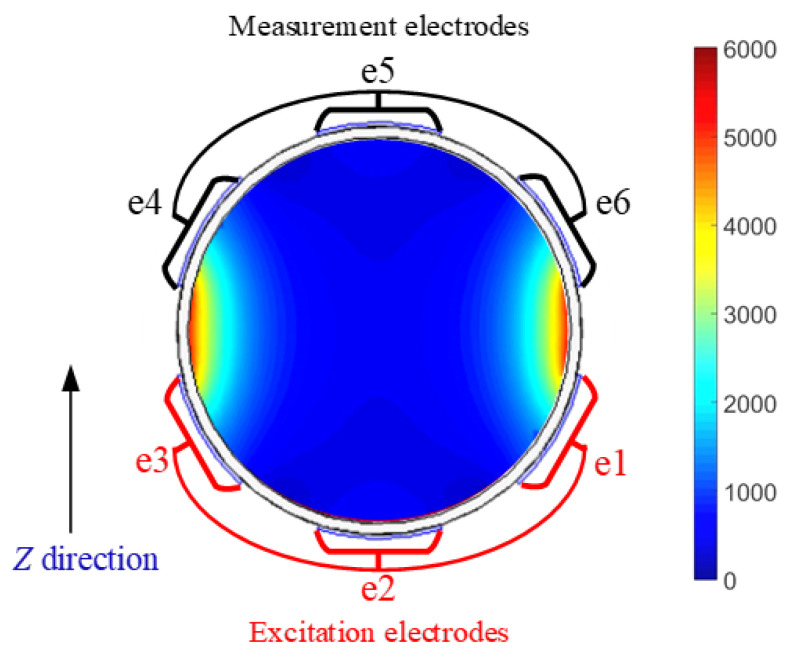
The sensitive field distribution of array capacitive sensors under multi-electrode excitation mode.

In the concave-ring excitation mode of the array capacitive sensor, the non-uniformity of sensitivity distribution is not conducive to solid concentration detection in the measurement field. Therefore, a multi-electrode excitation mode is designed based on the same array capacitive sensor to acquire global solid concentration, and this mode is specifically illustrated in Figure 3. As shown in Figure 3, three concave electrodes at the upper part and three at the lower part of the horizontal pipeline are respectively configured as electrode pairs in the multi-electrode excitation mode. The ring electrodes are grounded and act as guard electrodes to mitigate fringe effects and noise interference.

Figure 3 shows the sensitivity field distribution of the array capacitive sensor under the multi-electrode excitation mode. Compared to that of the concave-ring excitation mode, the sensitivity field distribution under the multi-electrode excitation mode exhibits greater uniformity in the measurement region, indicating that this mode is more suitable for global solid concentration detection. In addition, under this excitation mode, the left–right symmetry of the sensitivity field distribution is consistent with the symmetry of the particle distribution in the radial cross-section of the horizontal pipeline, which facilitates the measurement of particle concentration.

### 2.2. Structural Optimization of Array Capacitive Sensor

For the capacitive measurement system, excitation voltages applied to electrodes generate a steady electrostatic field within the detection domain. In the absence of a space charge inside the pipeline, the spatial potential distribution of the capacitive sensor satisfies the three-dimensional Poisson equation:

(1) $$\nabla \cdot \left[\varepsilon_{0}\varepsilon_{r}\left(x,y,z\right)\nabla \varphi \left(x,y,z\right)\right]=0$$

where φ(x,y,z) denotes spatial potential, $\varepsilon_{0}$ is vacuum permittivity, and $\varepsilon_{r}\left(x,y,z\right)$ represents the spatial relative permittivity distribution of the measured field. In the electrostatic measurement field, a higher local sensitivity is beneficial for measuring flow parameters in the local measurement region. Sensitivity represents the rate of capacitance change when the medium in the *k*th element (*k* = 1, 2, ..., *N*) switches from high to low permittivity, where *N* is the total number of elements. According to Geselowitz sensitivity theory [13,14], the sensitivity of the sensor to the *k*th element unit can be expressed as

(2) $$S_{i,j}\left(k\right)=-\int_{p(x,y,z)}\frac{E_{i}\left(x,y,z\right)\cdot E_{j}\left(x,y,z\right)}{U_{0}^{2}}\;dxdydz,\;i,j=1,2,\ldots ,L\;\mathrm{and}\;j\neq i$$

where U_0_ is the excitation voltage, and **E***_i_*(*x*,*y*,*z*) and **E***_j_*(*x*,*y*,*z*) are the electric field vectors at the *k*th element *p*(*x*,*y*,*z*) when U_0_ is applied to electrode *i* and electrode *j*, respectively. Typically, the *SVP* (Sensitivity Variation Parameter) described by Equation (3) is introduced to evaluate the uniformity of the sensitivity distribution in the measurement field [15].

(3) $$SVP_{i,j}=\frac{S_{i,j}^{dev}}{S_{i,j}^{avg}}\;i,j=1,2,\ldots L\;\mathrm{and}\;j\neq i$$

(4) $$S_{i,j}^{avg}=\frac{1}{m}\sum_{k=1}^{m}S_{i,j}\left(k\right)$$

(5) Si,jdev=1m−1∑k=1m[Si,j(k)−Si,javg(k)]212

Here, *m* is the number of discretized elements in the sensitive field, $S_{i,j}$(*k*) is the sensitivity of the *k*th element for the electrode pair *i*-*j*. $S_{i,j}^{avg}$ and $S_{i,j}^{dev}$ are the average sensitivity magnitude of all sensitive elements and the standard deviation of the sensitivity magnitude of all sensitive elements, respectively. A smaller SVP value indicates a more uniform sensitivity distribution in the measurement field. With sensitivity magnitude and uniformity as optimization objectives, orthogonal experiments are conducted via the numerical simulation model to optimize the structural parameters of the array capacitive sensor.

The numerical simulation model is established based on the finite element method (FEM). In this work, COMSOL Multiphysics 5.5 is utilized to build the steady-state electrostatic simulation model. Geometric bodies of electrodes, the pipeline and gas–solid two-phase media are established according to the dimensional specifications, and independent permittivity values are assigned to each geometric component to match their actual material characteristics. The excitation voltage applied to the electrodes is defined as the Dirichlet boundary condition to reproduce the electrostatic field distribution within the detection area. Specifically, the excitation voltage is set to 1 V, and the relative permittivities of the gas and pipeline are set to 1 and 3, respectively.

The optimized structural parameters of the sensor include the length of the concave electrode *L*_1_, the length of the ring electrode *L*_3_, the distance between the ring electrode and the concave electrode *L*_2_, and the wrap angle of the concave electrode θ. During the sensor structure optimization process, the optimization results of the array capacitive sensor structural parameters are quantitatively evaluated using the local mean sensitivity magnitude *S*-$S^{avg}$ and local sensitivity uniformity index *S*-*SVP* within the measurement region under the concave-ring excitation mode, as well as the global mean sensitivity magnitude *M*-$S^{avg}$ and global sensitivity uniformity index *M*-*SVP* within the measurement region under the multi-electrode excitation mode. Ultimately, the optimal structural parameter combination of the array sensor obtained from orthogonal experiments is shown in Table 1.

### 2.3. Hardware Measurement System

**Figure 4:**
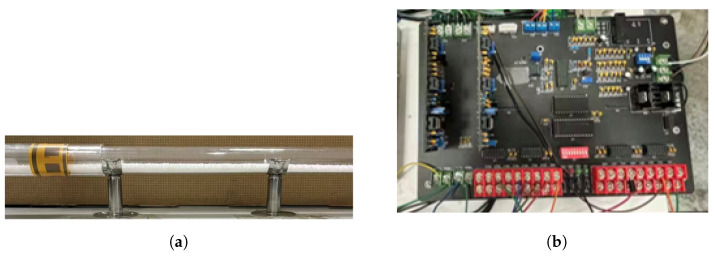
The hardware systems of the capacitive sensor and the measurement circuit (**a**) The array capacitive sensor (**b**) The measurement circuit.

An array capacitive sensor fabricated on a flexible PCB was installed on the horizontal pipeline of the laboratory gas–solid flows system, and a detection circuit was constructed to acquire measurement signals. Figure 4a shows the array capacitive sensor mounted outside the pipeline. Its electrode structural parameters are consistent with those of the simulation model. Figure 4b presents the detection circuit for the array capacitive sensor under the concave-ring excitation mode and multi-electrode excitation mode. During the experiments, Polyoxymethylene (POM) particles with a diameter of 2–3 mm and a relative permittivity of approximately 3.5 were used. In the measurement system, a sinusoidal signal with a frequency of 200 kHz and a peak-to-peak voltage of 5 V was applied to the excitation electrodes, while the measurement electrodes were connected to a module that converts capacitance values into voltage signals.

**Figure 5:**
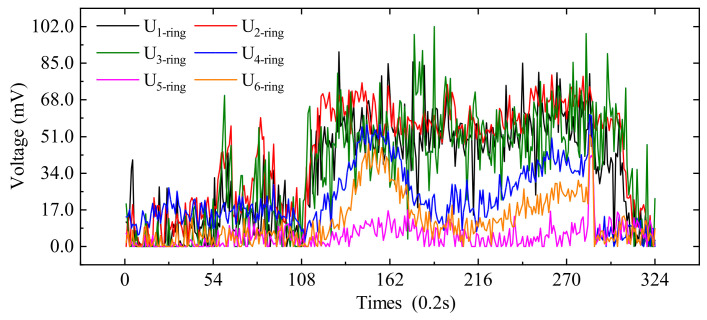
The measurement voltages of the array capacitive sensor obtained from the concave-ring excitation mode in the process of typical particle flow.

In a typical gas-solid flow process, the measurement signals acquired by the array capacitive sensor under the concave-ring excitation mode are shown in Figure 5. As can be seen from Figure 5, since particles are mainly concentrated at the bottom of the horizontal pipeline, the high particle concentration and low inter-particle porosity at the bottom result in larger measurement values for the concave electrode at the pipeline bottom, while the electrode at the pipeline top has a relatively smaller measurement signal amplitude. All the above phenomena indicate that the measurement signals of the array capacitive sensor under the concave-ring excitation mode can effectively reflect the distribution features of solid particles.

## 3. Coupling Simulation Model Based on CFD-DEM

### 3.1. Establishment of Gas–Solid–Electrostatic Coupling Field Model

Considering the complexity of gas–solid two-phase flow, it is difficult to obtain true instantaneous flow parameters in dynamic experiments. Although the accuracy of concentration measurements can be indirectly evaluated using mass flow parameters on a physical test platform, the calculation relies heavily on particle velocity. In horizontal pipelines, particle concentration is highly non-uniform across the radial cross-section, which makes accurate particle velocity measurement challenging with correlation-based methods and further compromises the effective validation of the solid concentration measurement method. Therefore, a 3D dynamic simulation model coupling gas–solid two-phase flow and electrostatic fields is established to quantitatively evaluate the proposed measurement method.

The coupling model framework adopted in this work originates from the experimentally verified model published in [16]. The Euler–Lagrange framework tracks discrete particle motion via DEM and solves continuous gas flow by CFD, taking particle–particle and particle–wall interactions into full consideration. Different from the Two-Fluid Model that requires complex closure equations for particle phases, this discrete particle method can accurately capture the micro spatial distribution of granular materials. Particle spatial positions extracted from the two-phase flow field are directly mapped to the electrostatic simulation domain to define regional permittivity distribution, realizing high-precision one-way coupling between gas–solid flow field and sensor electrostatic field.

Notably, the array capacitive sensor structure, CERM excitation mode and pipeline geometry validated experimentally in our earlier work [16] are consistent with the configuration adopted in the current study. A dedicated horizontal gas–solid two-phase experimental bench was constructed in the earlier work, and a series of static stratified flow experiments with different particle-stacking heights were carried out. By quantitatively comparing the capacitance variation trends, reconstructed particle distribution images and relative concentration errors obtained from simulation and physical tests, the accuracy and reliability of this coupling model for reproducing horizontal particle stratification distribution were fully validated. Based on this verified multi-field coupling model, diversified initial gas velocities and particle generation rates are set to generate multiple typical horizontal flow regimes, which serves as a reliable numerical tool for analyzing and validating the concentration measurement method proposed in this paper.

### 3.2. Simulation Parameter Configuration

A 3D dynamic simulation model coupling gas–solid two-phase flow and electrostatic fields is established, and the relevant parameters set in the simulation model are listed in Table 2. By adjusting parameters such as the particle generation rate and initial inlet gas velocity, simulation experiments involving various gas–solid flow processes are carried out. With this simulation model, the following data can be acquired: the measurement signals of the array capacitive sensor under the concave-ring excitation mode, the information of the particle distribution states, the measurement signals of the array capacitive sensor under the multi-electrode excitation mode, and the solid concentration within the corresponding measurement region.

## 4. Solid Concentration Inversion Methodology

Accurate characterization of the relationship between sensor signals and solid concentration in the measurement region is crucial for the accuracy of solid concentration measurements. When the sensitivity field is non-uniform, the fluctuation in the sensor signals caused by the changes in solid concentration exhibits nonlinear characteristics. This non-linearity can affect the accuracy of solid concentration measurement. To address this issue, the proposed method classifies the particle distribution features and converts the nonlinear measurement model into multiple linear measurement models to improve the measurement accuracy.

### 4.1. Particle Distribution Feature

Based on the coupling model of gas–solid two-phase flow fields and electrostatic field, three typical particle distribution cases inside horizontal pipelines are simulated in this study, covering flow regimes dominated by slug flow, dune flow, and suspended flow. Slug flow is characterized by granular material flowing in the form of slugs within the pipeline. Its distribution morphology can be divided into three regions: the slug front, middle, and rear. In the middle region, particles typically fill the pipeline, while the layer height at the front and rear gradually decreases at a certain inclined angle. During conveying, as the gas velocity increases gradually, the slugs tend to progressively collapse, leading to a gradual reduction in the height of the layered particle distribution on the radial cross-section. Dune flow refers to a dynamic flow state in which particles advance in a wavy manner in the flow, forming dune-like structures. The flow velocity and particle concentration directly affect the height and migration speed of dunes. Suspension flow develops when particles are uniformly dispersed in the fluid with minimal sedimentation at the pipeline bottom. This state typically forms under conditions of high flow velocity. From a macro perspective, significant differences exist among slug flow (Case 1), dune flow (Case 2), and suspended flow (Case 3) in horizontal pipelines. However, after segmenting various flow states into short-distance sections during the flow process, it can be considered that the particle distribution pattern mainly consists of particles accumulating at the pipeline bottom due to gravity and particles suspended in the upper part of the pipeline.

**Figure 6:**
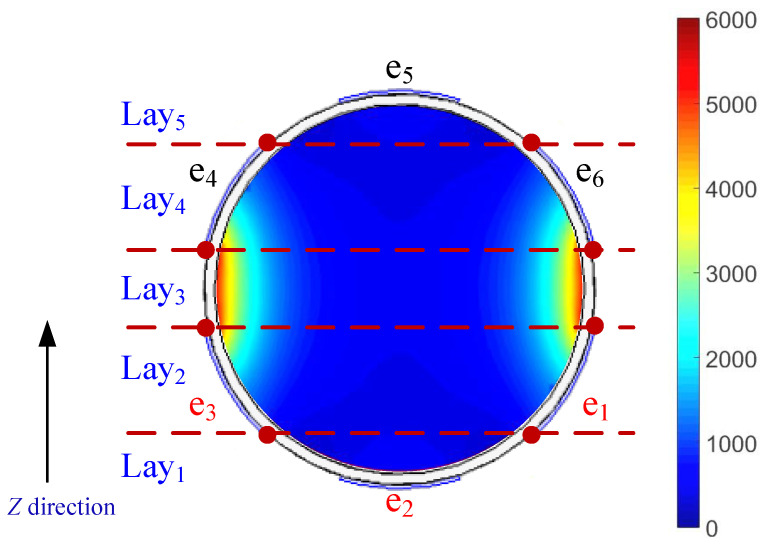
The division for the local layered region of ${\mathrm{Lay}}_{1}$∼${\mathrm{Lay}}_{5}$ in the measurement field.

**Figure 7:**
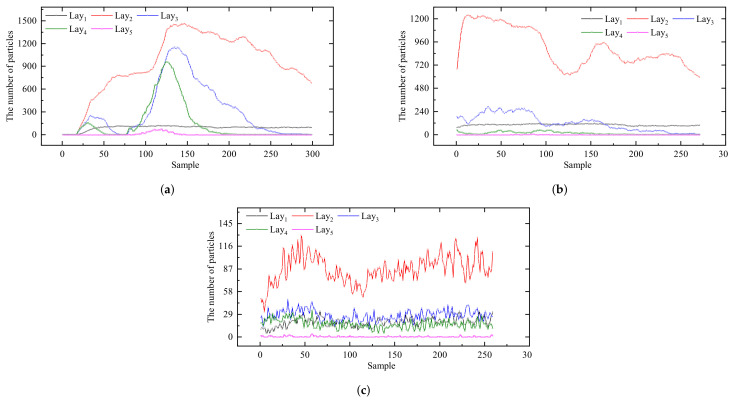
The variation in particle number for the local layered region during three typical gas–solid flow processes obtained from CFD-DEM numerical simulation (**a**) Case 1 (**b**) Case 2 (**c**) Case 3.

Additionally, according to the sensitivity field distribution characteristics under the concave-ring excitation mode and the multi-electrode excitation mode, the radial measurement cross-section is divided into five layered sensitive regions to study the particle distribution features, as illustrated in Figure 6, specifically including the first layer ${\mathrm{Lay}}_{1}$, the second layer ${\mathrm{Lay}}_{2}$, the third layer ${\mathrm{Lay}}_{3}$, the fourth layer ${\mathrm{Lay}}_{4}$, and the fifth layer ${\mathrm{Lay}}_{5}$. Correspondingly, Figure 7 shows the variation in particle number in the local layered regions of the conveying pipeline during three typical gas–solid flow processes simulated via CFD-DEM.

**Figure 8:**
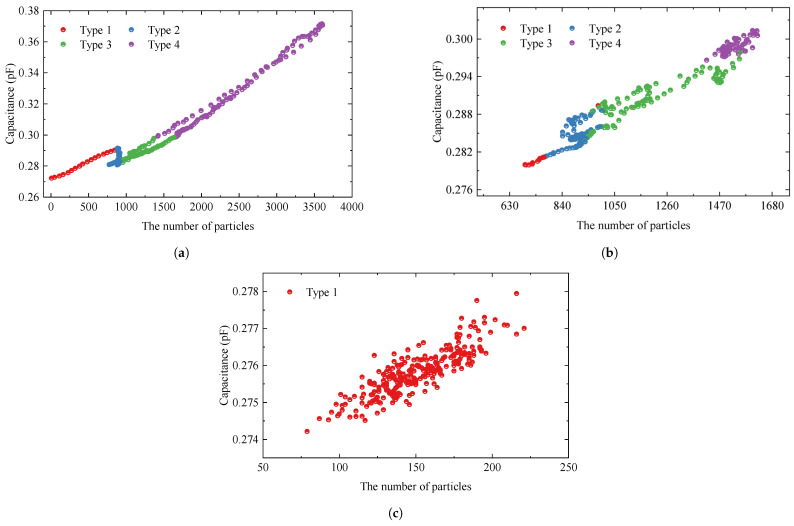
The relationship between measurements of the multi-electrode excitation mode and particles count of the measurement region during three typical gas–solid flow processes obtained from coupling simulation: (**a**) Case 1 (**b**) Case 2 (**c**) Case 3.

During gas–solid flows, the relationship between the measurement signals of the array capacitive sensor under the multi-electrode excitation mode and the number of solid particles in the measurement region is shown in Figure 8. Based on the linear correlation between the measurement signals under the multi-electrode excitation mode and solid concentration, the simulated local distribution characteristics of the gas–solid flows can be summarized into four typical types. When the ratios of particle numbers in layers ${\mathrm{Lay}}_{1}$∼${\mathrm{Lay}}_{5}$ to the total particle number and the total particle number inside the pipeline are selected as classification indicators, these two indicators can accurately characterize the spatial accumulation and overall transport intensity of particles on the cross-section of the pipeline. By setting reasonable thresholds, the local particle distribution can be divided into four typical types, with specific criteria as follows:

Type 1: Particles are highly accumulated in the layered region of ${\mathrm{Lay}}_{1}$ at the pipeline bottom, or suspended throughout the measurement area with a low overall solid concentration.

Type 2: Particles are mainly concentrated in the layered regions of ${\mathrm{Lay}}_{1}$ and ${\mathrm{Lay}}_{2}$.

Type 3: Particles are primarily concentrated in the layered regions of ${\mathrm{Lay}}_{1}$ and ${\mathrm{Lay}}_{2}$, while a portion of particles are also distributed in the layered region of ${\mathrm{Lay}}_{3}$.

Type 4: In addition to the particles distributed in the layered regions of ${\mathrm{Lay}}_{1}$ to ${\mathrm{Lay}}_{3}$, certain particles also exist in the layered regions of ${\mathrm{Lay}}_{4}$ or ${\mathrm{Lay}}_{5}$.

Through the above classification, the overall nonlinear measurement model can be decomposed into multiple linear models, as shown in Figure 8. However, further analysis suggests that in order to achieve higher fitting accuracy and reduce model errors, multiple linear fitting relationships can be further established within each distribution feature to more accurately characterize the relationship between measurement signals and solid concentration.

### 4.2. Feature Values Extracted from Measurement Signals

In this study, the concave-ring excitation mode is used to obtain particle distribution characteristics. Based on the measurement signals obtained from the concave-ring excitation mode, the specific feature parameters used to identify the particle distribution features include the following:

(1) Output values of the array capacitive sensor under the concave-ring excitation mode at the same time point.

Under the concave-ring excitation mode, the signal magnitudes in a complete measurement sequence from the array capacitive sensor can effectively reflect the spatial distribution characteristics within the local measurement region, especially the stratified distribution characteristics. To eliminate interference from external factors, all measured values are normalized using the empty-field and full-field information, as shown in Equation (6).

(6) $$x_{i}=\frac{x_{i}-x_{K}}{x_{M}-x_{K}}$$

where *x_M_* and *x_K_* denote the output values of the array sensor under concave-ring excitation mode when the measurement region is filled with solid medium and air, respectively. *x_i_* represents the measured output value under concave-ring excitation mode.

(2) Sample entropy values of measurement output under the concave-ring excitation mode at different time points.

In gas–solid two-phase flow, although the particle distribution state undergoes significant dynamic variations, such variation characteristics remain distinguishable within a specified time window. Therefore, the time series information of measurement signals can also be used to identify particle distribution features. The sample entropy of measurement signals at different time points is selected to extract the temporal features of the signals, which can effectively characterize the dynamic information of gas–solid flows. A small entropy value indicates that signal changes tend to be stable, usually corresponding to particles in a stable state of change within the local measurement area. For example, in relatively stable laminar flow or slug flow, the particle flow in local areas exhibits stable variations. A high entropy value indicates that particles are in a fluctuating state, where the particle flow in local areas shows strong fluctuations. In addition, sample entropy [17,18] is insensitive to data length and remains effective even for limited data sequences.

Given the collected raw data {$x_{j}$}, predefined pattern dimension *m*, and similarity tolerance *r*, the specific algorithm for sample entropy is as follows:

(a) Form the sequence {$x_{j}$} into *m*-dimensional vectors in order:

(7) $$X\left(i\right)=\left[x(i),x(i+1),\ldots ,x(i+m-1)\right],\;i=1,2,\ldots ,N-m+1$$

(b) Define the distance *d*[*X*(*i*), *X*(*j*)] between *X*(*i*) and *X*(*j*) as the maximum absolute difference of the corresponding elements:

(8) $$d\left[X\left(i\right),X\left(j\right)\right]=\max_{0∼m-1}\;\left|x\left(i+k\right)-x\left(j+k\right)\right|$$

Note that d[X(i),X(j)] is the maximum absolute difference between corresponding elements of X(i) and X(j). For each *i*, calculate d[X(i),X(j)] for all i=1,2,…,N−m+1.

(c) For a given threshold *r* (*r* > 0), count the number of *d*[*X*(*i*), *X*(*j*)] < *r* for each *i*. Then, calculate its ratio to the total number of vectors *N*-*m*, denoted as

(9) $$B_{i}^{m}\left(r\right)=\frac{1}{N-m}num\left\{d\left[X\left(i\right),X\left(j\right)\right]<r\right\},i=1,2,\ldots N-m+1,i\neq j$$

(d) The average value is denoted as

(10) $$B^{m}\left(r\right)=\frac{1}{N-m+1}\sum_{i=1}^{N-m+1}B_{i}^{m}\left(r\right)$$

(e) Increase dimension to *m* + 1. Repeat steps (a)–(d) to obtain $B_{i}^{m+1}\left(r\right)$ and $B^{m+1}\left(r\right)$.

(f) The theoretical sample entropy is

(11) $$SampEn\left(m,r\right)=\lim_{N\to \infty }\;\left\{-\ln\left[B^{m+1}\left(r\right)/B^{m}\left(r\right)\right]\right\}$$

For finite *N*, the estimated sample entropy is

(12) $$SampEn\left(m,r,N\right)=-\ln\left[B^{m+1}\left(r\right)/B^{m}\left(r\right)\right]$$

The value of *SampEn*(*m*, *r*, *N*) depends on parameters *m*, *r*, and *N*. Different embedding dimensions *m* and similarity tolerances *r* yield different sample entropy values. Generally, *m* = 1 or 2 and *r* = 0.1–0.25$SD_{x}$ produce sample entropy with reasonable statistical properties. And $SD_{x}$ denotes the standard deviation of the original time series data {$x_{j}$}.

(3) Signal vertical stratification ratio under concave-ring excitation mode

To more accurately describe the differences in particle spatial distribution under the same particle distribution feature and establish measurement models with stronger adaptability, the signal vertical stratification ratio is defined as a key parameter. This parameter can directly reflect the vertical distribution uniformity and relative accumulation position of particles across the pipeline radial cross-section, which is defined as follows:

(13) $$R=\frac{x_{bot1}+x_{bot2}+x_{bot3}}{x_{top1}+x_{top2}+x_{top3}}$$

where $x_{\mathrm{top}1}$, $x_{\mathrm{top}2}$, and $x_{\mathrm{top}3}$ are the measurement signals corresponding to the three upper electrodes at the top of the pipeline under concave-ring excitation mode, while $x_{\mathrm{bot}1}$, $x_{\mathrm{bot}2}$ and $x_{\mathrm{bot}3}$ are the signals corresponding to the three lower electrodes at the bottom of the pipeline under concave-ring excitation mode.

**Figure 9:**
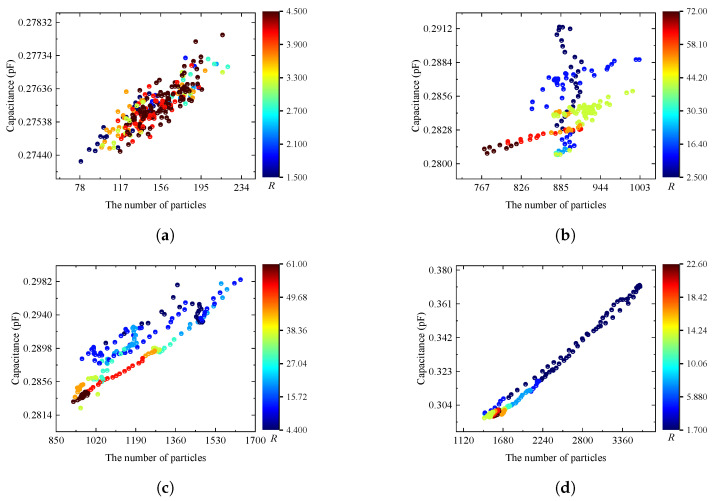
Distribution of *R* values of measurement samples under multi-electrode excitation for four types of distribution features obtained from coupling simulations: (**a**) Type 1; (**b**) Type 2; (**c**) Type 3; (**d**) Type 4.

When particles are mainly deposited at the bottom of the pipeline, the measurement signals of the bottom three electrodes are significantly stronger than those of the top three electrodes, resulting in a high value of parameter *R*. As particles diffuse upward, the signals of the top three electrodes gradually increase, and thus *R* decreases accordingly. Figure 9 illustrates the *R* values of measurement samples under multi-electrode excitation for the four types of distribution features derived from the simulated samples described in Section 4.2. As shown in Figure 4, using the *R* value enables effective clustering and partitioning of the relationship between measurement signals and solid concentration within the same particle distribution feature, providing a reliable basis for the subsequent construction of refined multi-linear measurement models.

### 4.3. Particle Distribution Feature Recognition Based on BP-Adaboost

Ensemble learning is a technique that enhances model performance and generalization ability by combining multiple base models. It generates the final prediction result by weighting or voting on the outputs of multiple models, thereby reducing the bias and variance of a single model and improving the robustness and generalization ability of the overall model [19,20]. Two commonly used techniques for building ensemble classifiers are Boosting and Bagging, and AdaBoost is a widely adopted algorithm in the Boosting family. This learning approach places greater focus on misclassified samples during training and adaptively adjusts the weights of these samples after each Boosting iteration. Specifically, in each iteration, the weight of training samples misclassified by the current base classifier is increased to prioritize them in the next iteration, while the weight of correctly classified samples is reduced accordingly. By repeating this process, the algorithm gradually focuses on the most challenging samples until the number of weak classifier training iterations reaches a predefined threshold, at which point the entire learning process is completed and the loop terminates.

Numerous studies have demonstrated that various classification models can serve as weak classifiers within the AdaBoost framework [21]. Notably, the selection of weak classifier type and its parameters is critical for optimizing the performance of the AdaBoost algorithm. In this study, a Back-Propagation (BP) neural network [22] is used as the weak classifier in the AdaBoost algorithm. By virtue of the iterative weighting mechanism of AdaBoost, the BP-AdaBoost algorithm focuses on hard-to-classify samples, performs weighted fusion on multiple trained BP weak classifiers, and ultimately constructs a high-precision strong classifier. It fully combines the excellent nonlinear fitting capability of BP neural networks with the prominent generalization improvement characteristics of AdaBoost. The specific framework of the BP-AdaBoost algorithm is shown in Algorithm 1.
**Algorithm 1:** Flow of BP-AdaBoost algorithm
 
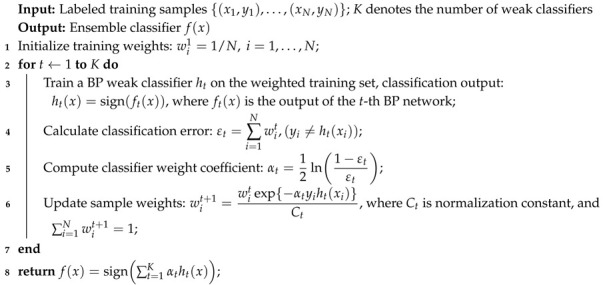



### 4.4. Multi-Linear Concentration Measurement Model Based on K-Means Clustering

On the basis of the recognition of particle distribution features, the measurement signals and solid concentration within the same category still exhibit different variation trends, making it difficult for a single linear model to achieve accurate fitting. To address this issue, the K-means clustering algorithm is introduced in this study to perform refined grouping of samples within the same category.

As a classical unsupervised learning algorithm based on distance criteria, K-means clustering aims to partition samples into K clusters with similar characteristics through iterative optimization, minimizing feature differences within each cluster and maximizing discrepancies between different clusters. The algorithm uses Euclidean distance as the similarity metric, and its objective function is to minimize the sum of squared distances from each sample to its corresponding cluster center, which is expressed as follows:

(14) $$J=\sum_{i=1}^{K}\sum_{x\in T_{i}}{∥R_{x}-\mu_{i}^{R}∥}^{2}$$

where *K* is the preset number of clusters, $T_{i}$ denotes the sample set belonging to the *i*-th cluster, $R_{x}$ represents the clustering feature value corresponding to sample *x*, and $\mu_{i}^{R}$ is the centroid of clustering feature values in the *i*-th cluster. The term ${∥R_{x}-\mu_{i}^{R}∥}^{2}$ characterizes the deviation between the clustering feature value of an individual sample and its cluster centroid, and the double summation computes the total intra-cluster deviation loss *J*.

In the modeling process, the signal vertical stratification ratio *R* is used as the clustering feature for the samples under the same particle distribution feature. This parameter can effectively characterize the spatial distribution differences of sensor signals and serves as a key indicator for distinguishing sub-patterns within the same particle distribution category.

After clustering, a quadratic polynomial fitting model between the measurement signals under the multi-electrode excitation mode and solid concentration is established. The quadratic polynomial can be expressed as

(15) $$Y_{fit}=p_{1}X^{2}+p_{2}X+p_{3}$$

where *X* is the measurement signal obtained from multi-electrode excitation mode, $Y_{fit}$ is the predicted solid concentration, and $p_{1},p_{2},p_{3}$ are the fitting coefficients.

The trained cluster centers and the fitting coefficients for each linear measurement model are obtained and stored during the calibration stage. For a new sample to be measured, its corresponding cluster is determined by the minimum distance criterion based on the Euclidean distance between the feature value and each cluster center:

(16) $$cluster\_id=\arg\min_{i}\left|R_{new}-\mu_{i}^{R}\right|$$

where $\mu_{i}^{R}$ is the cluster center, and $R_{new}$ is the signal vertical stratification ratio *R* of the measured sample. The quadratic fitting model corresponding to the determined cluster is then invoked to complete the concentration prediction. Compared with the single global linear model, K-means clustering decomposes the complex single relationship into multiple simple piecewise relationships through data-driven adaptive grouping. This improves the fitting accuracy and generalization ability of the measurement model, and ultimately achieves the high-precision inversion of solid concentration.

## 5. Results and Performance Analysis

This section quantifies the overall performance of the proposed method using validated simulation data. Section 5.1 defines the evaluation metrics adopted for quantitative analysis. Section 5.2 and Section 5.3 share the same simulation dataset, sequentially presenting particle distribution classification performance and corresponding concentration measurement accuracy.

### 5.1. Performance Indicators

The performance of the solid concentration measurement method proposed in this paper is quantitatively evaluated from two aspects: the measurement error of solid concentration and the accuracy of identifying the particle distribution features. The relative measurement error (Re) parameter shown in Equation (17) is used to evaluate the measurement error between the true solid concentration and the measured solid concentration.

(17) $$Re=\frac{|\beta_{ms}-\beta_{ts}|}{\beta_{ts}}\times 100\%$$

where $\beta_{ms}$ is the measured solid concentration, and $\beta_{ts}$ is the true solid concentration.

Two measurement parameters are adopted to evaluate the accuracy of particle distribution feature classification: per-class accuracy and overall accuracy. Overall accuracy can intuitively reflect the general classification performance of the model on the entire dataset, while per-class accuracy is capable of revealing the model recognition capability for each category. In particular, it can expose the deficiencies of the model in handling minority classes or hard-to-classify categories.

The formula for calculating the parameter of per-class accuracy is

(18) $$AS=\frac{N_{TS}}{N_{AS}}\times 100\%$$

where $N_{TS}$ represents the number of correctly classified samples for a type of particle distribution feature; $N_{AS}$ represents the total number of samples for a type of particle distribution feature.

The parameter of overall accuracy is calculated as:

(19) $$AA=\frac{N_{TA}}{N_{AA}}\times 100\%$$

where $N_{TA}$ represents the number of correctly classified samples across all distribution features; $N_{AA}$ represents the total number of samples across all distribution features.

### 5.2. BP-Adaboost Classification Performance Evaluated on Simulation Samples

In the experiments, the BP-AdaBoost algorithm is employed to classify the particle distribution features using the simulation dataset. This dataset contains 800 samples, including 277 samples for Type 1, 173 for Type 2, 187 for Type 3, and 163 for Type 4. The dataset is divided into a training set and a test set with a ratio of 60% to 40%, respectively. In addition, the sample distribution characteristics of the dataset are similar to those of actual industrial production processes, where the probability of occurrence of different particle distribution features differs substantially, resulting in a natural imbalance in sample collection.

On the premise that the algorithm performance is satisfactory, the classification algorithm adopts default toolbox configurations to guarantee stable algorithm performance and simple operation. The single BP base classifier has a single hidden layer with six neurons, the maximum training iteration is set to 10, the learning rate is 0.1, and the MSE training target is 0.00004. Each binary sub-ensemble contains three base classifiers with standard AdaBoost weight iteration rules. For the randomly selected test set, the average classification accuracy for each type is 94.5%, and the overall classification accuracy is 95%, which verifies that the BP-AdaBoost algorithm has excellent classification performance.

### 5.3. Concentration Inversion Accuracy Analysis Under Matched Classification Results

Furthermore, during the measurement, the corresponding linear measurement model for the identified particle distribution feature is adaptively matched by calculating the Euclidean distance between the signal vertical stratification ratio parameter *R* and each cluster center. The cluster centers and their corresponding linear measurement models are predetermined in the calibration stage using the K-means clustering algorithm and the training set. Considering both fitting accuracy and model complexity, the number of clusters for Types 1–3 is set to 3, while that for Type 4 is fixed at 2. All other algorithm parameters follow default toolbox configurations: squared Euclidean distance as the distance metric, one replicate per clustering run, empty clusters filled with singleton clusters generated from the farthest global sample, and a maximum iteration limit of 100.

**Figure 10:**
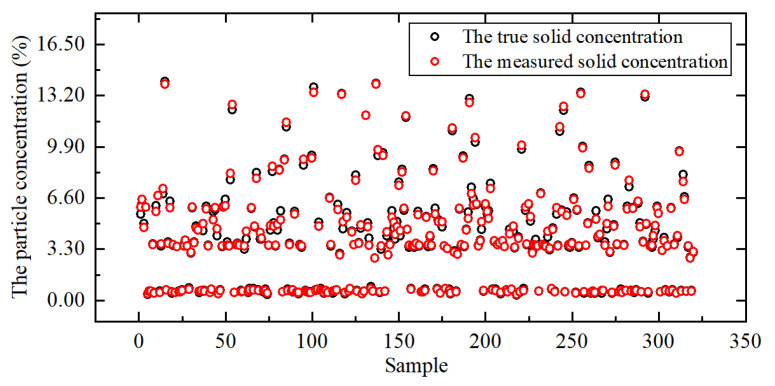
The true concentration and the measured concentration obtained from the proposed measurement method.

Through the synergistic cooperation of the two algorithms, the nonlinear mapping relationship between the sensor signal and the concentration can be decomposed into multiple linear relationships, thereby effectively suppressing measurement errors caused by uneven particle distribution and improving the accuracy of concentration inversion. For the randomly selected test set, the average value of relative measurement error Re and its standard deviation obtained by the proposed method are 4.41% and 0.0478, respectively, within the solid concentration range of 0.34–14.08%. Figure 10 presents the true particle concentration values and the measured particle concentration values of the test samples. The true solid concentration, regarded as reference values, derived from validated CFD-DEM simulation model, and the measured solid concentration is predicted by the proposed measurement method. It can be observed from Figure 10 that the error between the measured values and the true values is small.

To compare the performance of representative measurement approaches reviewed in the Introduction, a multi-dimensional comparison is summarized in Table 3, including the application scenarios of the designed methods, sensor hardware configuration, online computational cost, and measurement accuracy. As listed in Table 3, each existing method has evident application limitations. The dual-helical capacitive sensor optimized for more uniform sensitivity serves as the control group under consistent horizontal gas–solid flow conditions in this study. The test results reveal its measurement error is far higher than that of the proposed measurement method. The electrostatic-capacitance dual-sensor fusion scheme requires two complete sets of hardware, bringing higher cost and computational load. ECT focuses on flow cross-section visualization with heavy reconstruction computation rather than high-precision concentration quantification.

Owing to inconsistent concentration test ranges and error evaluation criteria across the above references, direct error comparison is not rigorous. Nevertheless, the proposed method maintains stable high precision across a broad concentration range (0.34–14.08%) covering slug, dune and suspended flows, whereas most alternative schemes are verified within narrow concentration ranges or limited flow regimes. Overall, the proposed measurement method achieves a better trade-off between low hardware cost, low computational overhead, wide measurable range and strong adaptability to various particle distribution.

## 6. Conclusions

This paper proposes a solid concentration measurement method for horizontal gas–solid flows based on an adaptive model matching strategy with array capacitive sensor. Different from studies proposing new sensing physical mechanisms or original basic machine learning algorithms, this work integrates mature capacitive detection, BP-Adaboost and K-means clustering to form a dedicated measurement framework for horizontal gas–solid flows. Using a single array capacitive sensor, the concave-ring excitation mode and the multi-electrode excitation mode are used to capture the information of local particle distribution and solid concentration, respectively. Firstly, based on the linear correlation between multi-electrode excitation signals and solid concentration, the particle distribution features are divided into four typical patterns, and the classification criterion refers to the particle proportion of layered distribution in the measurement area. The BP-AdaBoost algorithm is introduced to realize accurate classification of distribution features, and the average per-class accuracy reaches 94.5% on the random test set.

For an identified particle distribution pattern, the measurement model is adaptively matched by calculating the Euclidean distance between the signal vertical stratification ratio and cluster centers, so as to realize the inversion of the solid concentration. Multiple linear measurement models between multi-electrode excitation signals and solid concentration are constructed by K-means clustering in the calibration stage. The experimental results show that the proposed method can adapt to complex and variable particle distribution states. Within the solid concentration range of 0.34–14.08%, the average relative measurement error is 4.41%, which can effectively improve the detection accuracy of solid concentration in horizontal gas–solid two-phase flow systems. Benefiting from the customized dual-excitation electrode mode and adaptive model matching mechanism, the proposed method exhibits prominent advantages in concentration measurement accuracy and adaptability for horizontal gas–solid two-phase flow.

The present study mainly validates the concentration measurement method via numerical simulation, yet the dynamic physical experiment verification is restricted without synchronous flow velocity reference data. Therefore, we will integrate real-time flow velocity acquisition modules into the measurement platform to complete more sufficient dynamic experimental tests for fully verifying the practicability of the proposed method. Furthermore, the BP-Adaboost classification and K-means matching algorithm used in this work exhibit limited stability and classification precision under extreme stratified flow states. Future research will focus on developing optimized intelligent algorithms with stronger anti-interference capabilities to further boost the robustness of local solid concentration measurement.

## Figures and Tables

**Table 1:** Structural parameters of simulation model of the array capacitive sensor.

*R*_1_	*R*_2_	*L*_4_	*L*_5_	*L*_1_	*L*_3_	*L*_2_	θ
18 mm	21 mm	10 mm	10 mm	*R*_1_	0.3*R*_1_	0.3*R*_1_	35°

**Table 2:** Parameters of the simulation model of gas–solid two-phase flow based on CFD-DEM.

	Solid Particle		Gas Phase	
Geometry	Shape	Spherical	Gas material	Air
	Particle diameter	1.6 mm	Gas density	1.205 kg/m^3^
	Particle material	Plastic pellet	Gas viscosity	1 × 10^−5^ kg·m^−1^·s^−1^
Particle density		1000 kg/m^3^	Boundary conditions	Inlet: velocity-inlet
				Output: outflow
				Wall: no-slip
Shear modulus		3.759 × 10^7^ Pa	Initial value for inlet	8 m/s
Poisson ratio		0.33	Time step	0.0001 s
			Discretization schemes	Implicit
Particle to particle	Coefficient of restitution	0.8	Pipe Shape	3D cylinder
	Coefficient of static friction	0.3	Pipe Direction	horizontal
	Coefficient of rolling friction	0.01	Pipe Length	1.5 m
Particle to Wall	Coefficient of restitution	0.45	Inner Pipe diameter	36 mm
	Coefficient of static friction	0.5	Pipe Thickness	3 mm
	Coefficient of rolling friction	0.05	Pipe material	Acrylic plastic
Particle number		12,000	Pipe density	1200
Time step		2.5 × 10^−6^ s	Shear modulus	8.889 × 10^8^ Pa
			Poisson ratio	0.35

**Table 3:** Performance comparison of measurement methods for horizontal gas–solid two-phase flow.

Measurement Method	Application Scenarios	Sensor Hardware Configuration	Online Computational Cost	Measurement Accuracy
Proposed method in the study	Accurately measure horizontal gas–solid flow concentration across various flow regimes.	Single array capacitive sensor with dual excitation modes	Signal feature extraction, feature recognition and linear arithmetic operation	The average relative error is 4.41% for the solid concentration range of 0.34–14.08% under slug flow, dune flow, and suspended flow.
Dual-helical capacitive sensor (control group in this work)	Optimized for uniform sensitivity to measure concentration of dense-phase pneumatic pulverized coal conveying [10].	Single pair of helical electrodes and a single excitation mode	Only linear arithmetic operation	The average relative error is 11.45% for the same test cases as those in this work.
Electrostatic-capacitance multi-sensor fusion system [11,12]	Obtain dual-component volume concentration under biomass/coal/air three-phase conveying.	Independent electrostatic sensor and array capacitive sensor, dual hardware synchronous acquisition	Signal feature extraction, feature recognition and linear arithmetic operation	Ref. [11] covers roping and stratified flows; concentration range: 0.41 × 0.1–2 × 0.1% (coal), 2.4 × 0.1–11.8 × 0.1% (biomass). Maximum absolute errors: 0.07 × 0.1% (coal), 0.18 × 0.1% (biomass). Ref. [12] covers rope, dispersed and stratified flows; concentration range: 0.1046–0.4468‰ (coal), 0.1363–0.5386‰ (biomass). Average relative errors: 1.5% (coal), 2.2% (biomass).
Electrical Capacitance Tomography (ECT) [5]	For two-phase flow cross-section distribution visualization.	Single array capacitive sensor with full-electrode excitation modes	Iterative image reconstruction	Four flow patterns such as plug flow, fluidized flow, slug flow and stratified flow were observed in the horizontal conveying.

Note: Data for the proposed method and double-helical sensor are from the coupled simulations; the parameters of other approaches are extracted from the references reviewed in the Introduction.

## Data Availability

No data was used for the research described in the article.
